# P-1082. A Multidrug-Resistant Organism Tracking and Alert System is Associated with Lower Methicillin Resistant Staphylococcus aureus Infection Rates at Department of Veterans Affairs Medical Centers

**DOI:** 10.1093/ofid/ofaf695.1277

**Published:** 2026-01-11

**Authors:** Amanda Vivo, Cara Ray, Geneva Wilson, Reside Jacob, Makoto Jones, Natalie Hicks, Christopher D Pfeiffer, Charlesnika T Evans

**Affiliations:** CINCCH at Hines VA, Hines, IL; VA Center of Innovation for Complex Chronic Healthcare, Hines, Illinois; VA Center of Innovation for Complex Chronic Healthcare, Hines, Illinois; VA Center of Innovation for Complex Chronic Healthcare, Hines, Illinois; VA Salt Lake City Health Care System, Salt Lake City, Utah; National Infectious Diseases Service, Specialty Care Services, Veterans Health Administration, Washington DC, District of Columbia; VA Portland Health Care System, Portland, Oregon; Northwestern University and VA, Hines, Illinois

## Abstract

**Background:**

Early identification of patients admitted with multidrug resistant organisms (MDROs) is important for preventing transmission. The Department of Veterans Affairs (VA) Bug Alert (VABA) notifies infection prevention personnel at VA Medical Centers (VAMCs) when a patient with an MDRO history is admitted to acute or long-term care. Here we investigate the association of VABA use with MRSA healthcare-associated infection (HAI) rates in VAMCs.

**Methods:**

The was a cross-sectional study of VABA utilization May 1, 2022-December 22, 2023. Facility-level MRSA HAI rates and bed days of care (BDOC) were obtained from the VA Inpatient Evaluation Center. MRSA HAI was defined as infection ≥ 3 days after admission per CDC/NHSN definition. Facilities were categorized as high, medium, or low complexity based on patient characteristics and clinical or teaching programs. The relationship between days of VABA use by VA staff and MRSA HAI rates was assessed using Pearson’s correlation coefficient. A two-part finite mixture model was used to analyze the relationship between MRSA HAI rate and days of VABA use, adjusted for facility complexity level.

**Results:**

There were 280 MRSA HAIs from 108 VAMCs, where 73% of VAMCs were high complexity, 14.8% were medium, and 12.2% were low complexity. The mean number of days of VABA use was 19, 25, and 56 days in low, medium, and high complexity facilities respectively. Days of usage ranged from 0-484. The average MRSA infection rate was 0.10 per 1000 BDOC. After controlling for facility complexity, for each one-day increase in VABA use there was a decrease in infection rate of 0.002 MRSA cases per 1000 BDOC (p-value=0.01).

**Conclusion:**

Increased VABA use was associated with decreased MRSA HAI rates after adjusting for differences in facility complexity. This underscores the potential of this tool as part of comprehensive infection control strategies. Further investigation of its impact on other MDROs and feasibility and benefits of expanding VABA implementation to non-VA facilities is warranted.

**Disclosures:**

Christopher D. Pfeiffer, MD, MHS, Department of Defense/MedPace: Grant/Research Support

